# Asthmatic children that are uncontrolled despite inhaled corticosteroids have a distinct breathprint (the pacman2 study)

**DOI:** 10.1186/2045-7022-5-S2-O6

**Published:** 2015-03-23

**Authors:** Susanne Vijverberg, Paul Brinkman, Jan Raaijmakers, Kors van der Ent, Peter Sterk, Anke-Hilse Maitland-van der Zee, Leo Koenderman

**Affiliations:** 1 Utrecht University, Pharmaceutical Sciences, Utrecht, Netherlands; 2 AMC, University of Amsterdam, Respiratory Medicine, Amsterdam, Netherlands; 3 Wilhelmina Children's Hospital, UMCU, Pediatric Respiratory Medicine, Utrecht, Netherlands; 4 UMCU, Respiratory Medicine, Utrecht, Netherlands

## Background

Measuring patterns of volatile organic compounds ('VOCs') in exhaled breath (so-called 'breathprint') is a novel metabolomic non-invasive approach to study molecular signatures of respiratory disease.

## Aim

to study whether controlled and uncontrolled asthmatic children treated with inhaled corticosteroids (ICS) can be identified according to their breathprint.

## Method

VOCs were measured in thirty-three asthmatic children (age: 11.8 yrs, SD 2.3 yrs) who participated in the PACMAN2 study. All children were current users of ICS. Current asthma control was assessed using the Asthma Control Questionnaire. Long-term asthma control was based on reported symptoms in the four seasons preceding baseline and follow-up visit. A single vital capacity volume of exhaled air was collected upon 5 minutes of normal breathing through a three-way non-re-breathing valve with a VOC filter in a Tedlar bag. The VOCs in the breath sample were subsequently captured on Tenax GR Tubes and analyzed offline on a validated panel of four eNoses with different sensor technologies (Owlstone Lonestar, Sensigent Cyranose 320, Comon Invent eNose and Tor Vergata TEN). Breathprints were analysed per eNose using principal component analyses (PCA). ROC curves and cross-validated accuracy values of significant (t-test) principal components were used to assess the accuracy of the devices to discriminate between controlled and uncontrolled patients. Additionally, the fraction of exhaled nitric oxide (FeNO) was measured with a NIOX Mino and an expiration time of 6 seconds.

## Results

Two eNoses in the panel were able to discriminate current uncontrolled asthmatic children and controlled asthmatic children according to their breathprint (accuracy: 69.7-75.8%; AUC: 0.71-0.74). Three eNoses were able to identify long-term uncontrolled asthmatic children and long-term controlled asthmatic children (accuracy: 66.9-87.5%; AUC: 0.81-0.97). For both outcomes the Comon Invent, a metal oxide sensor, was most accurate in separating both groups. In contrast, FeNO was a poor marker to discriminate between controlled and uncontrolled asthmatic children (AUC: 0.56, respectively 0.61).

## Conclusion

Breathprint analyses can accurately distinguish uncontrolled from controlled asthmatic children. In contrast, a single FeNO measurement cannot. These preliminary results suggest that breathprints might be able to identify distinct childhood asthma phenotypes.

